# Uninterpretable Dynamic Susceptibility Contrast-Enhanced Perfusion MR Images in Patients with Post-Treatment Glioblastomas: Cross-Validation of Alternative Imaging Options

**DOI:** 10.1371/journal.pone.0136380

**Published:** 2015-08-21

**Authors:** Young Jin Heo, Ho Sung Kim, Ji Eun Park, Choong-Gon Choi, Sang Joon Kim

**Affiliations:** 1 Department of Radiology and Research Institute of Radiology, Asan Medical Center, University of Ulsan College of Medicine, Seoul, Republic of Korea; 2 Department of Radiology, Inje University, Busan Paik Hospital, Busan, Republic of Korea; University of Nebraska Medical Center, UNITED STATES

## Abstract

**Purpose:**

The purpose of this study was to evaluate the accuracy of diffusion-weighted imaging (DWI) and dynamic contrast-enhanced (DCE) perfusion MR imaging for distinguishing tumor recurrence from post-treatment effect as alternatives to dynamic-susceptibility contrast-enhanced (DSC) perfusion MR imaging when the DSC image is uninterpretable.

**Materials and Methods:**

This retrospective study was approved by our institutional review board. Seventy one post-treatment glioblastoma patients who showed enlarged contrast-enhancing lesions on follow-up MR images after concurrent chemoradiotherapy and uninterpretable DSC images for corresponding enhancing lesions, underwent additional DWI and DCE MR imaging. The primary outcome was the frequency of interpretable DWI and DCE MR cases in these 71 patients. The secondary outcome was the area under the receiver operating characteristic curve (AUC) of DWI and DCE imaging parameters for distinguishing tumor recurrence from post-treatment effect in selected patients with interpretable DWI and DCE images. The imaging parameters were quantified as 10% cumulative histogram cutoff of apparent diffusion coefficient (ADC10) and 90% cumulative histogram cutoff of initial area under the time signal intensity curve (IAUC90). The AUCs were cross-validated by using leave-one-out method.

**Results:**

Of the 71 patients, the uninterpretable DSC images were associated with treatment-related hemorrhage within the corresponding enhancing lesions (n = 54, 76.1%) and a near skull base location (n = 17, 23.9%). The frequencies of interpretable DWI and DCE image were 51 (71.8%) and 59 (83.1%) of the 71 cases with uninterpretable DSC images, respectively. Of the 45 selected patients with interpretable DWI and DCE images, the combination of DWI with DCE imaging showed a superior diagnostic performance than DWI or DCE imaging alone for differentiating tumor recurrence from post-treatment effect (cross-validated AUC: 0.78 versus 0.55 and 0.73 for reader 1; cross-validated AUC: 0.78 versus 0.53 and 0.75 for reader 2, respectively). Cross-validated accuracy of the single and combined imaging parameters also showed the highest for the combination of DWI with DCE MR imaging (72.9% for reader 1; 72.5% for reader 2) and the lowest for DWI alone (54.0% for reader 1; 56.4% for reader 2). Inter-reader agreement for DCE imaging was higher than that for DWI (intraclass correlation coefficient: 0.95 versus 0.87).

**Conclusion:**

DCE MR imaging could be a superior and more reproducible imaging biomarker than DWI for differentiating tumor recurrence from post-treatment effect in patients with post-treatment glioblastoma when DSC MR images are not interpretable.

## Introduction

Differentiation of tumor recurrence from treatment-related change in post-treatment glioblastoma remains a diagnostic challenge due to the similar, contrast-enhanced MR imaging features caused by blood-brain barrier (BBB) disruption. Previous studies have shown that dynamic susceptibility contrast-enhanced (DSC) perfusion MR imaging is strongly correlated with tumor angiogenesis and can reliably predict tumor recurrence [1,2]. It is also easily obtained within a few minutes using commercially available software. Therefore, DSC perfusion MR imaging has been widely used as a diagnostic tool for post-treatment glioblastoma in order to differentiate tumor recurrence from treatment-related change [2–5].

However, DSC perfusion MR imaging has some inherent limitations. This method could be susceptible to the magnetic field inhomogeneity caused by hemorrhage, calcification, and surgical materials [3]. If a lesion is located near the skull base, it appeared as marked signal loss and geometric distortion [2,3,6,7]. Such signal loss on the gradient echo is the result of intravoxel dephasing caused by the local susceptibility gradient [8]. Marked signal loss and geometric distortion interferes with the accurate estimation of DSC perfusion MR parameters. A previous study reported that 7% of their patients were excluded due to image distortion and the susceptibility artifact in the DSC perfusion MR images [3]. DSC perfusion MR imaging analyzed T2 and T2* changes and parameters can be extracted when contrast agent remains within the intravascular space. In cases of post-treatment glioblastoma, contrast material extravasates into the tissue due to disruption or absence of a blood-brain-barrier (BBB). This will then cause a biasing, T1-based contrast enhancement and extra T2*shortening in the tissue. Moreover, the BBB breakdown-related contrast agent leakage depends on the tumor cell density and tumor cell distribution within the extravascular space, thereby resulting in an additional susceptibility calibration factor [9].

On the other hand, DCE perfusion MR imaging could be less sensitive to magnetic field inhomogeneity than DSC perfusion MR imaging. It provides better spatial resolution, thus allowing accurate characterization of microvascular structures within a tumor [1,4,5]. Current studies have demonstrated that it is a reliable method for assessing the vascular microenvironment, guiding the biopsy, and differentiating treatment-related change from tumor recurrence [10,11]. Diffusion-weighted imaging (DWI) basically derived from spin-echo echo-planar imaging sequences has shown a higher signal-to-noise ratio and less susceptibility artifact than DSC perfusion MR imaging [12]. A previous study [13] found that spin-echo, echo-planar sequences, such as DWI, are less sensitive to hemorrhagic lesion than the gradient-recalled, echo-planar sequence. This can be explained in part by a spin-echo 180° refocusing pulse before the application of oscillatory gradient pulses. These results agree with those of a previous study in which the gradient-recalled, echo echo-planar sequence and the gradient-recalled, echo sequence were much more sensitive than the spin-echo, echo-planar sequence or other fast spin-echo sequences for depicting chronic hemorrhage [14].

Our hypothesis is that DCE perfusion MR imaging and DWI can provide additional information when DSC perfusion MR images show signal loss and geometric distortion which make it difficult to differentiate tumor recurrence from treatment-related change. Therefore, the purpose of this study was to assess the utility of DWI and DCE perfusion MR imaging for distinguishing tumor recurrence from post-treatment effect in selected glioblastoma patients with uninterpretable DSC perfusion MR images.

## Materials and Methods

### Study patients

The Institutional Review Board of Asan Medical Center approved this retrospective study and waived the need for written informed consent from the participants. The patient records / information was anonymized and de-identified prior to analysis. A retrospective review of 755 post-treatment glioblastoma patients who underwent follow-up MR imaging from October 2006 to August 2014, selected 71 patients according to the following criteria: (a) who had been histologically proven as having glioblastoma before concurrent chemoradiotherapy (CCRT); (b) who had received CCRT following surgery; (c) who had newly appeared or gradually enlarged, contrast-enhancing lesions seen on subsequent follow-up MR imaging; (d) who had uninterpretable DSC perfusion MR images with signal loss and geometric distortion for corresponding enlarged, contrast-enhancing lesions; and (e) who underwent DWI and DCE MR imaging for their enlarged, contrast-enhancing lesions during the same imaging session as that of DSC MR imaging.

**Fig 1:**
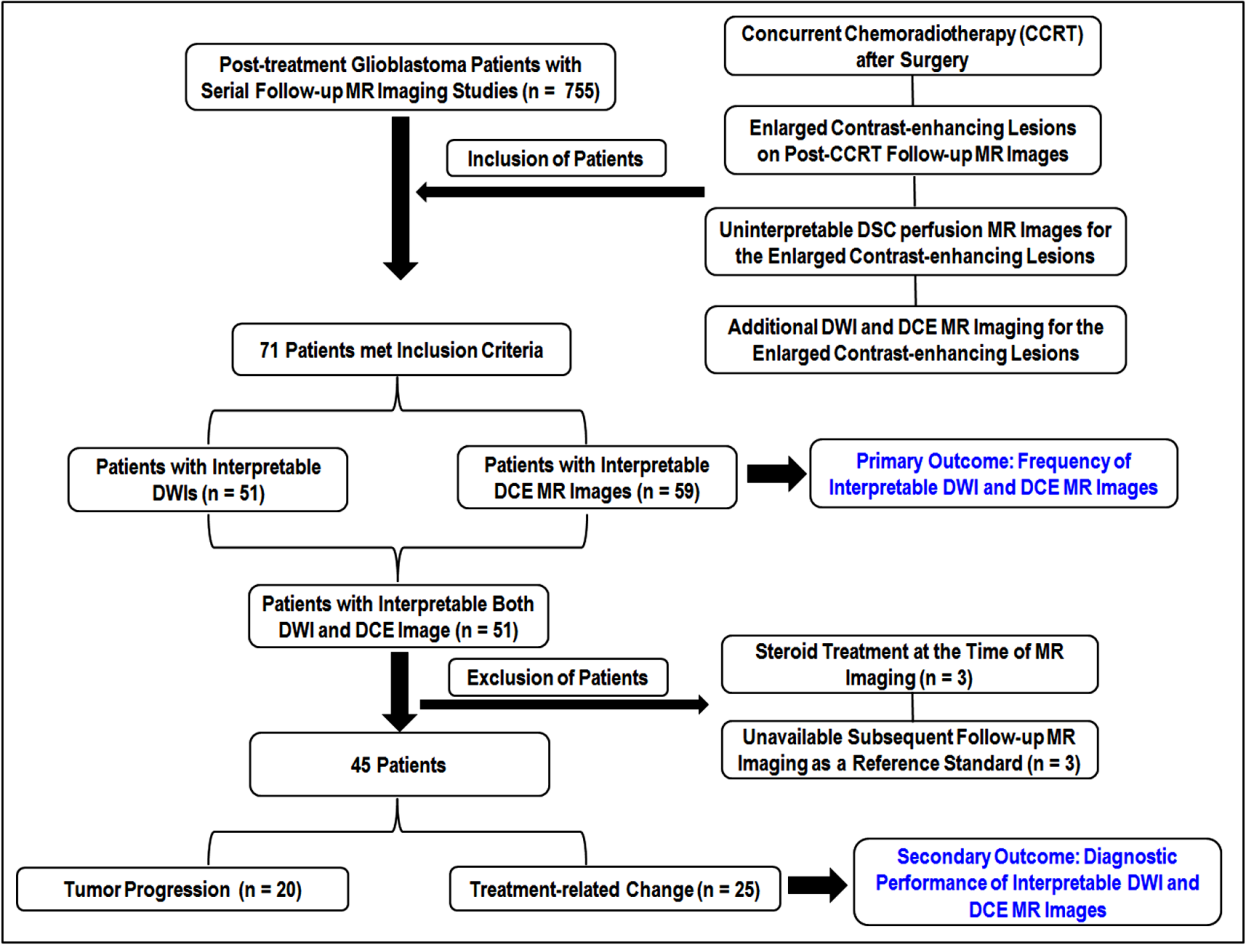
The diagram of the study patients’ accrual process.

The primary outcome of this study was the frequency of interpretable DWI and DCE MR image in these 71 patients with uninterpretable DSC perfusion MR image. The interpretable image quality was defined as the images without any signal loss and geometric distortion for the corresponding enlarged contrast-enhancing lesions and it was approved by a clinically experienced neuroradiologist based on the findings of T1-, T2- and T2*-weighted images within corresponding enhancing lesions. Among the 71 patients with uninterpretable DSC images, 51 patients showed interpretable image quality on both DWI and DCE MR image. Of these 51 patients, three who had received steroids at the same time as DSC and DCE perfusion MR imaging, were excluded. In the remaining 48 patients, three had unavailable subsequent follow-up MR imaging as a reference standard for the diagnostic performance of DWI and DCE imaging. We finally selected 45 patients (21 men and 24 women; mean age, 53.9 years; range, 27–73 years) in order to determine the diagnostic performance of DWI and DCE MR imaging when DSC MR images were not interpretable. The secondary outcome of this study was the area under the receiver operating characteristic curve (AUC) of DWI and DCE imaging parameters for distinguishing tumor recurrence from post-treatment effect in these selected 45 patients. None of them were treated with another therapeutic agent, such as bevacizumab, before MR image acquisition. The study patients’ inclusion and exclusion process is shown in Fig 1.

### Final diagnosis as the reference standard

Nine patients who underwent second-look surgery were classified with tumor recurrence (n = 6) and treatment-related change (n = 3). The pathologic features of tumor recurrence and treatment-related change were markedly different. Well-known pathologic findings of tumor recurrence included the presence of cellular sheets and atypical cells with mitotic features. Geographically coagulative necrosis, reactive vascular changes, vascular hyalinization, vascular necrosis, perivascular chronic inflammation, dystrophic calcification, and gliotic change were considered as treatment-related change.

Clinicoradiologic diagnosis was achieved as an alternative method when second-look surgery could not be performed. Tumor recurrence (n = 14) and treatment-related change (n = 22) were determined by agreement of the neurosurgeon and the neuroradiologist after complete review of both the clinical information and the MR imaging findings. If there was a decrease in size or stabilization of the contrast-enhancing lesions at least six months after the completion of CCRT, it was considered as treatment-related change. If contrast-enhancing lesions were progressively enlarged on serial follow-up MR imaging with correlated neurologic deterioration, they were diagnosed as tumor recurrence [4,15].

### Imaging protocol

A 3T MR system (Achieva; Philips Medical Systems, Best, The Netherlands) with an 8-channel, sensitivity-encoding head coil was used to acquire all of the MR images. Acquisition of MR imaging was as follows: T2-weighted imaging, fluid-attenuated inversion recovery (FLAIR) imaging, DWI, pre-contrast T1-weighted imaging, DCE perfusion MR imaging, contrast-enhanced T1-weighted MR imaging, and then DSC perfusion MR imaging. DCE perfusion MR imaging is obtained before DSC perfusion MR imaging in order to minimize the T1- leakage effect and the T2/T2* residual effects by preloading the contrast agent [2, 16–18].

DSC perfusion MR imaging using the gradient-echo, echo-planar sequence was acquired during the administration of a standard dose of 0.1 mmol/kg of gadoterate meglumine using a power injector (Spectris; Medrad, Pittsburgh, PA, USA) at a rate of 4 mL/sec and immediately followed by a 20-mL, continuous saline flush at the same injection rate. The detailed parameters of this sequence are as follows: TR/TE, 1808/40 msec; flip angle, 35°; FOV, 24 cm; slice thickness/gap, 5 mm/2 mm; matrix, 128 x 128; and the total acquisition time was one minute and 54 seconds. DSC perfusion MR imaging was acquired with complete tumor volume coverage and the same section orientations as conventional MR imaging.

DWI was performed in three, orthogonal directions and was then combined into a trace image. The DWI parameters were as follows: repetition time (TR)/echo time (TE), 3000/56 msec; diffusion gradient encoding, *b* = 0 and 1000 sec/mm^2^; FOV, 25 cm; slice thickness /gap, 5 mm/2 mm; matrix, 256 × 256; and acquisition time, 39 seconds. Apparent diffusion coefficient (ADC) maps were acquired from DWI data at b values of 0 and 1000 sec/mm^2^.

DCE perfusion MR imaging was acquired using 3-dimensional gradient echo imaging and 21 slices were achieved before, during, and after injection of gadoterate meglumine (Dotarem; Guerbet, Paris, France) with both a standard dose (0.1mmol/kg) and rate (4 mL/s). The dynamic acquisition was performed with a temporal resolution of 3.22 seconds, and contrast was administrated after 10 baseline dynamics (total: 120 dynamics). DCE perfusion MR images were acquired with the following parameters: TR/TE, 6.4/3.1 msec; flip angle, 15°; FOV, 24 cm; slice thickness, 4 mm with no gap; matrix, 184 x 186; and total acquisition time, five minutes and 41 seconds.

### Image processing

For quantitative analysis of diffusion and perfusion MR imaging, all of the imaging data were transferred to a personal computer. In-house software based on Analysis of Functional Neuroimages (AFNI) software (National Institute of Mental Health, Bethesda, MD, USA) was used for the processing of the ADC and perfusion parametric maps [19].

For temporal smoothing, the dynamic time signal intensity (SI) curve was smoothed using low-pass filter, to reduce effects of noise and spikes in the dynamic SI curve. This smoothing does not affect spatial resolution but may reduce the ability to detect rapid signal changes. Use of temporal smoothing may improve significantly the quality of the curve fitting procedure used to obtain estimate for the DCE kinetic parameters. The SI normalization was done compared to its baseline SI value on the time–SI curves. The baseline of all data was set at zero. We then calculated the initial area under the time SI curve (IAUC) at 30 sec. Determination of IAUC at 30 sec was based on a previously published study suggesting that the initial contrast agent wash-in of tumor recurrence was usually achieved within 30 seconds after contrast agent arrival [4].

For DSC perfusion MR imaging, the relative cerebral blood volume (CBV) was calculated after elimination of contrast recirculation and contrast leakage correction using numeric curve integration. All of the pixels from the entire relative CBV map were then normalized relative to the mean value of the region-of-interest (ROI) placed on the contralateral, normal-appearing white matter, and thus resulting in a normalized CBV (nCBV) map.

### Image analysis

**Fig 2:**
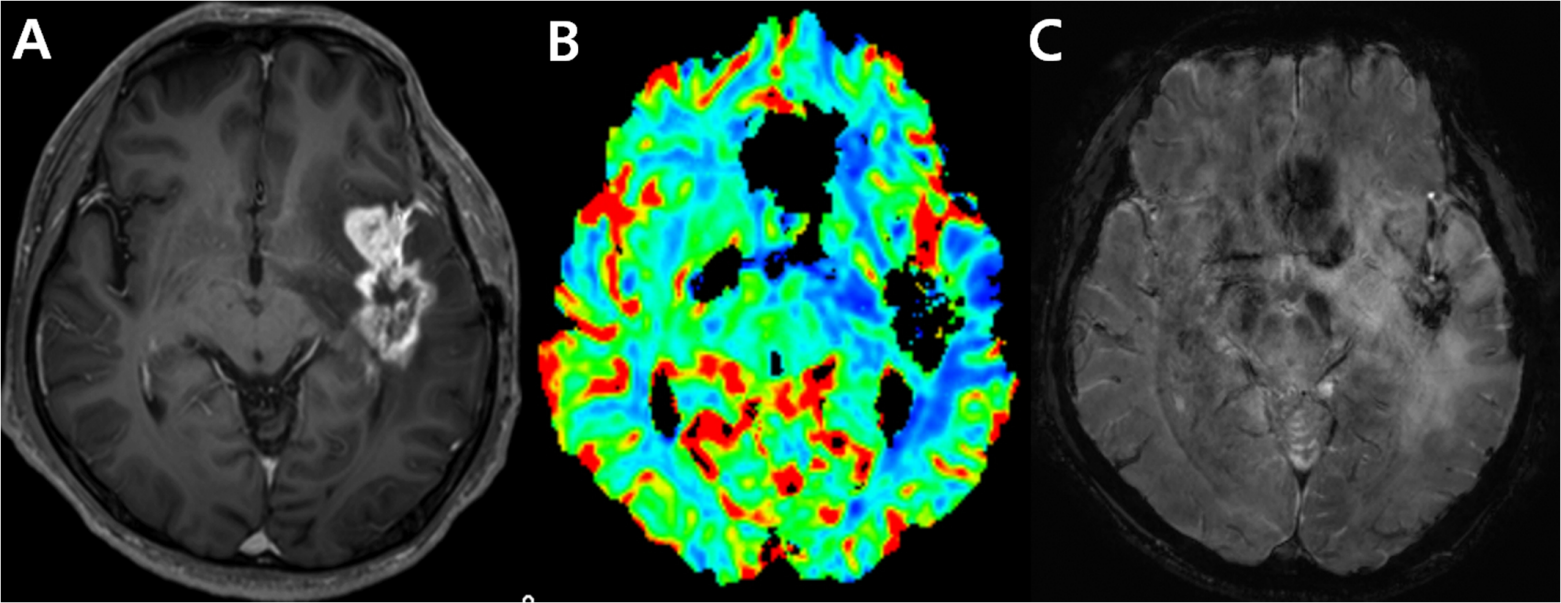
An example of an uninterpretable DSC perfusion MR image. Images obtained in a 56-year-old man clinicoradiologically considered as having tumor progression. Contrast-enhanced, T1-weighted image (A) acquired 19 weeks after concomitant chemoradiotherapy (CCRT) shows an enhancing lesion in the temporal lobe. DSC perfusion MR image (B) shows signal loss in the corresponding contrast-enhancing lesion due to treatment-related hemorrhage confirmed on susceptibility weighted image (C).

Geometrical distortions and signal loss causing marked image degradation on the DSC perfusion MR imaging corresponding to contrast-enhancing lesions were considered as uninterpretable DSC MR images [1,7,20]. Such lesions have a nil value of nCBV. An example of uninterpretable DSC perfusion MR image is shown in Fig 2.

For the co-registration between contrast-enhanced T1-weighted imaging and DCE MR imaging or DWI, an automatic coregistration was first used by the use of mutual information. If the coregistration quality was insufficient, additional manual coregistration was performed by two readers. All transformations, i.e. translations, rotations, and scales, applied during the manual coregistration were applied only to the overlay image (DCE image or DWI).

The two readers who were blinded to the clinical outcome, then independently segmented the entire contrast-enhancing lesion volumes from the regions of macroscopic necrosis, cystic areas, and vessels by drawing ROIs on the co-registered, contrast-enhanced, T1-weighted images. The segmentation quality was approved by the clinically experienced neuroradiologist. Finally, the two readers calculated the 90th percentile cumulative histogram cutoffs for IAUC (IAUC90) and the 10th percentile cumulative histogram cutoff for ADC (ADC10) from the segmented entire contrast-enhancing lesion volumes. The 90th and 10th percentile cumulative histogram cutoffs which are less influenced by random statistical fluctuations than the maximum and minimum values, respectively, have been validated and optimized in previously published studies [4,15].

### Statistical analysis

The Student’s t-test was used to determine the difference of the DWI and DCE MR imaging parameters between the tumor progression and post-treatment effect groups. The sensitivity and specificity of the imaging parameters to distinguish tumor recurrence from post-treatment effect, were calculated using the receiver operating characteristic (ROC) curve analysis. The area under the ROC curve (AUC) and the accuracy of the combined DWI and DCE MR imaging parameters were cross-validated by using a leave-one-out cross-validation.

A leave-one-out cross-validation was used to assess whether the single and combined imaging parameters could be used to correctly classify as tumor recurrence or post-treatment effect. Using this method, one subject was selected as the validating set and the other subjects were classified as the training set. Such procedures were repeated until every case was selected as the validation set.

The intraclass correlation coefficient (ICC) was used to compare the ADC values calculated from DWI and IAUC calculated from DCE by the two readers.

Commercially available software (SPSS, version 21.0, SPSS, Chicago, IL, USA) and R version R 2.15.3 (R Project for Statistical Computing, http://www.r-project.org) were used for the statistical analyses. A significant *P* value difference was determined as less than .05. Multiple comparisons were controlled using Bonferroni Correction.

## Results

### Frequency of interpretable DWI and DCE image in patients with uninterpretable DSC image

The uninterpretable DSC images were associated with macro-hemorrhages which were confirmed on T1-, T2- and T2*-weighted images within corresponding enhancing lesions (n = 54, 76.1%) and proximity to the skull base in the frontal and temporal lobes (n = 17, 23.9%). Of the 71 cases with uninterpretable DSC images, the frequency of interpretable DWI and DCE image were 51 (71.8%) and 59 (83.1%), respectively, whereas 20 DWIs and 12 DCE images were also uninterpretable. All of the patients with interpretable DWI also showed interpretable DCE images, therefore the frequency of interpretable DWI and DCE image was 51 (71.8%). Of the 20 uninterpretable DWIs, 14 cases have contrast-enhancing lesion with proximity to the skull base and 6 cases showed macro-hemorrhage in the corresponding enhancing lesions. Of the 12 uninterpretable DCE images, all cases showed macro-hemorrhage in the corresponding enhancing lesions.

### Diagnostic performance of IAUC90 and ADC10

Of the 51 patients with uninterpretable DSC images and interpretable DWI and DCE image, 45 selected patients were assessed for the diagnostic performance of DWI and DCE image after exclusion of 6 patients due to steroid treatment at the time of imaging and loss of follow-up studies. Of the 45 study patients, 20 (44.4%) were finally diagnosed with tumor recurrence and the remaining 25 (55.6%) with post-treatment effect on pathologic or clinic-radiologic follow-up results as a reference standard. Descriptive statistics regarding the clinical parameters obtained in each group are summarized in Table 1.

**Table 1:** Comparison of the Study Patient Demographic Data.

Variables	Tumor Recurrence (n = 20)	Post-treatment effect (n = 25)
No. of male patients	11(55.0%)	10 (40.0%)
No. of female patients	9 (45.0%)	15 (60.0%)
Age (years)^a^	52.4 ± 11.7	55.3 ± 11.9
Second-look surgery	6 (30.0%)	3 (12.0%)
Mean radiation dose (at CCRT, Gy)	58.1	59.3
Mean time interval between CCRT and an enlarging, contrast-enhancing lesion (Weeks)	14.7 ± 10.6	13.3 ± 12.1

Abbreviations: Gy = gray.

^a^Data are mean ± standard deviation.

**Fig 3:**
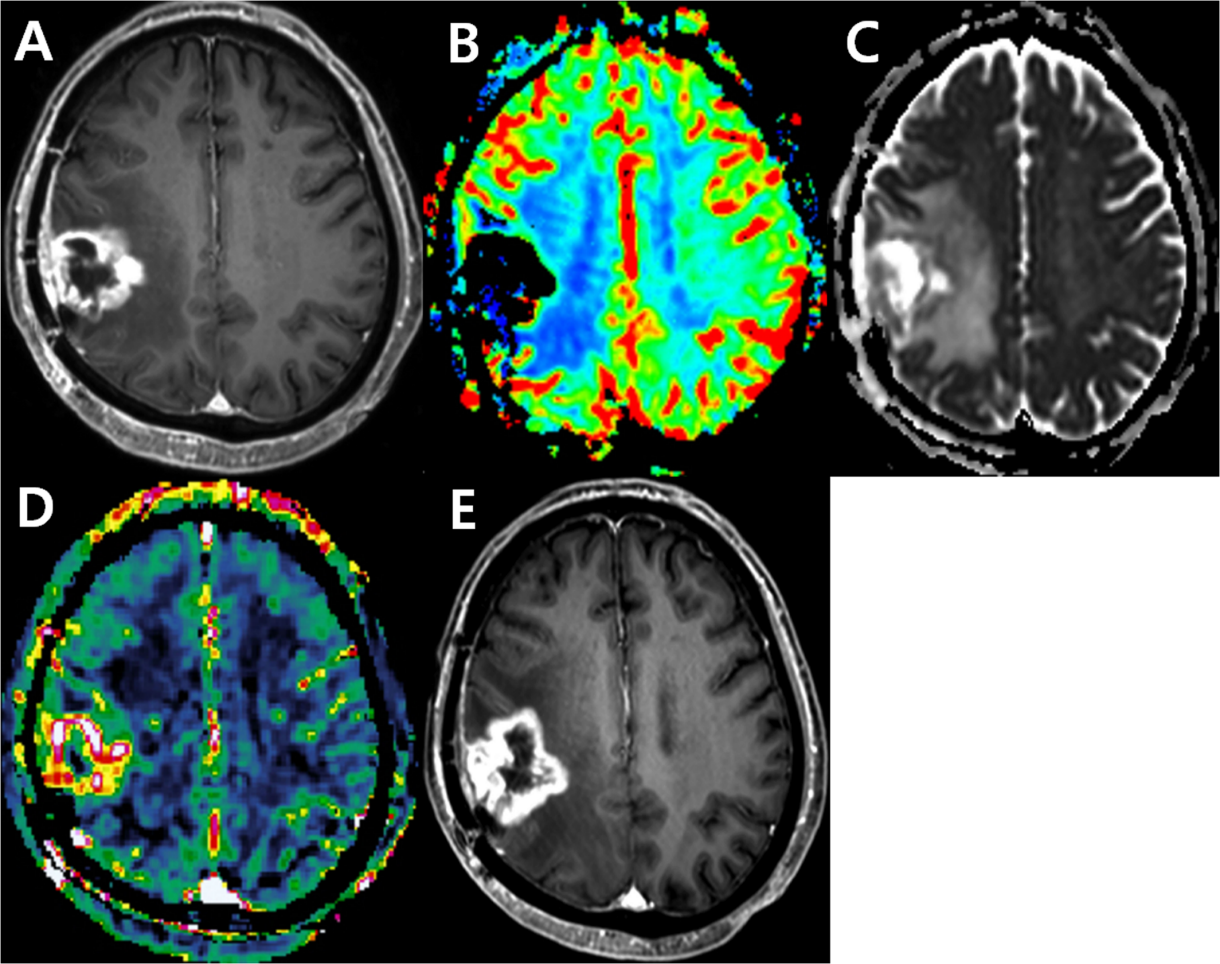
Images obtained in a 72-year-old man clinicoradiologically considered as having tumor progression. Contrast-enhanced, T1-weighted image (A) acquired 14 weeks after concomitant chemoradiotherapy (CCRT) shows a rim-enhancing lesion in the right hemisphere. DSC perfusion MR image (B) shows signal loss in the corresponding area possibly caused by treatment-related hemorrhage. The corresponding contrast-enhancing solid tumor portion shows intermediate to low ADC value (C) as well as increased permeability on the DCE perfusion MR image (D). Contrast-enhanced, T1-weighted image (E) acquired 22 weeks after CCRT shows the contrast-enhancing lesion is more enlarged, suggesting tumor progression.

**Fig 4:**
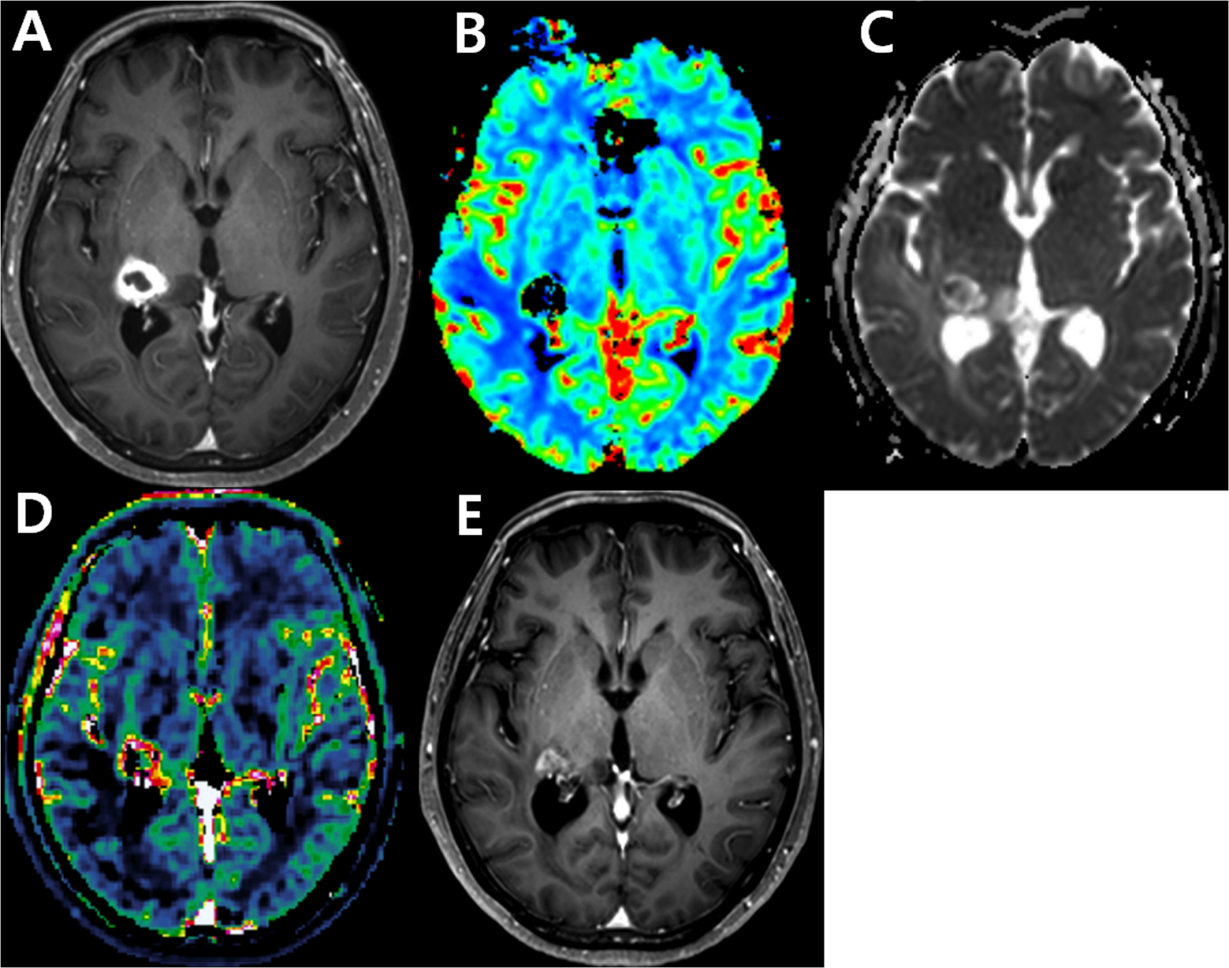
Images obtained in a 60-year-old woman clinicoradiologically considered as having treatment-related change. Contrast-enhanced, T1-weighted image (A) acquired 14 weeks after concomitant chemoradiotherapy (CCRT) shows a rim-enhancing lesion in the right thalamus. DSC perfusion MR image (B) shows signal loss in the corresponding area possibly caused by treatment-related hemorrhage. The corresponding contrast-enhancing solid tumor portion reveals high ADC value (C) as well as increased permeability on the DCE perfusion MR image (D). Contrast-enhanced, T1-weighted image (E) acquired 19 weeks after CCRT shows the contrast-enhancing lesion is stabilized, suggesting treatment-related hemorrhage.

IAUC90 was differed significantly between tumor recurrence and treatment-related change (*P* = .007 for reader 1; *P* = .005 for reader 2, respectively). However, ADC10 did not differ significantly between the two groups for either reader (P >.025 for both readers). Table 2 shows the sensitivity and specificity using IAUC90 and ADC10 to differentiate tumor recurrence from post-treatment effect in post-treatment glioblastoma patients with uninterpretable DSC images. The sensitivity and specificity were 80.0% and 72.0% for reader 1 and 70.0% and 84.0% for reader 2, respectively. The cases of tumor progression and post-treatment effect are presented in Figs 3 and 4.

**Table 2:** Comparison of the Imaging Parameters between the Tumor Recurrence and the Treatment-related Change in Patients with Uninterpretable DSC MR Images.

Reader and final diagnosis	ADC10 (x10^-3^mm^2^/s)	IAUC90
Reader 1		
Tumor recurrence	0.96± 0.14	8.64 ± 3.96
Post-treatment effect	0.99 ± 0.19	5.84 ± 2.63
*P*-value	> .025	.007
Sensitivity	65.0%	80.0%
Specificity	64.0%	72.0%
Reader 2		
Tumor recurrence	0.96 ± 0.15	8.81 ± 3.84
Post-treatment effect	0.99 ± 0.21	5.95 ± 2.62
*P*-value	>.025	.005
Sensitivity	65.0%	70.0%
Specificity	52.0%	84.0%

Abbreviations: ADC10 = 10th percentile cutoff value of ADC, and IAUC90 = 90th percentile cutoff value of IAUC.

*P*-values were calculated using the Student t-test

For the multiple comparisons of the imaging parameters, a *P*-value < .025 indicates a significant difference.

Table 3 shows the cross-validated AUC and accuracy of the single and combined imaging parameters in post-treatment glioblastoma patients with uninterpretable DSC images. In order to differentiate tumor recurrence from post-treatment effect, DCE perfusion MR imaging showed a higher cross-validated AUC than DWI (AUC: 0.73 versus 0.55 for reader 1; 0.75 versus 0.53 for reader 2, respectively) (Table 3)(S1 Fig). The combination of DWI with DCE imaging showed a superior diagnostic performance than DWI or DCE imaging alone for differentiating tumor recurrence from post-treatment effect (cross-validated AUC: 0.78 versus 0.55 and 0.73 for reader 1; cross-validated AUC: 0.78 versus 0.53 and 0.75 for reader 2, respectively). Cross-validated accuracy of the single and combined imaging parameters also showed the highest for the combination of DWI with DCE MR imaging (72.9% for reader 1; 72.5% for reader 2) and the lowest for DWI alone (54.0% for reader 1; 56.4% for reader 2).

**Table 3:** Leave-one-out, Cross-validation Results of the Single and Combined Imaging Parameters in Patients with Uninterpretable DSC MR Images.

Reader and imaging method	Cross-validated AUC	Cross-validated accuracy
Reader 1		
DWI	0.55	54.0%
DCE MR imaging	0.73	67.3%
DWI + DCE imaging	0.78	72.9%
Reader 2		
DWI	0.53	56.4%
DCE MR imaging	0.75	65.8%
DWI + DCE imaging	0.78	72.5%

Abbreviations: AUC = area under the receiver operating characteristic curve.

### Interreader agreement

Inter-reader agreement for the measurement of IAUC90 (ICC, 0.95) was higher than for the measurement of ADC10 (ICC, 0.87).

## Discussion

In this study, we reviewed uninterpretable DSC perfusion MR images in patients with post-treatment glioblastomas in order to determine their causes. Treatment-related hemorrhage and proximity to skull base might be associated with signal loss and geometric distortion. We found that DWI images were interpretable in 71.8% and DCE MR images were interpretable in 83.1% of the patients with uninterpretable DSC perfusion MR images. Uninterpretable DWIs were mainly associated with having contrast-enhancing lesion with proximity to the skull base and all of uninterpretable DCE images had macro-hemorrhages in the corresponding enhancing lesions. DCE MR imaging showed a higher diagnostic accuracy and substantially better inter-reader agreement than DWI for differentiating tumor recurrence from post-treatment effect in selected post-treatment glioblastoma patients with uninterpretable DSC MR images. Moreover, The combination of DWI with DCE imaging showed a superior diagnostic performance than DWI or DCE imaging alone for differentiating the two groups.

DWI, DSC MR imaging, and DCE MR imaging reflect different tumor pathophytsiology as imaging biomarkers and might provide compensatory information for brain tumor biology and treatment response. However, there has been the lack of combined clinical applications of DSC and DCE MR perfusion imagings in brain tumor study. In our selected cases with uninterpretable DSC perfusion MR images, both DWI and DCE MR imaging provided added values in terms of both interpretability and diagnostic performance. Nevertheless, DSC MR image has different information about tumor vascularity such as tumor vessel density. In terms of practicability, DSC MR imaging needs very short acquisition time and does not need complex pharmacokinetic modeling, compared with DCE MR image. Moreover, DSC MR image could provide a higher specificity for differentiating tumor recurrence from treatment-related effect in the previous study [15]. Therefore in our MR protocol for brain tumor, DSC MR imaging has been a routine sequence for tumor perfusion. However, according to our results, DWI and DCE MR imaging could be optional methods in selected patients with uninterpretable DSC images and might impact on the proposed change to the clinical practice. More specifically, DCE MR images could be a better alternative method in case that uninterpretable DSC images for contrast-enhancing lesion are mainly associated with proximity to the skull base.

To our knowledge, no previous study has considered the added value of DCE perfusion MR imaging and DWI to uninterpretable DSC perfusion MR imaging in patients with post-treatment glioblastomas. Our study evaluated the diagnostic accuracy of DWI and DCE perfusion MR imaging parameters in selected patients with uninterpretable DSC perfusion MR images. We used IAUC90 which is one of the model-free pharmacokinetic DCE MR imaging parameters and has been validated in the previous studies for differentiating recurrent tumor from treatment-related change [4,15]. Narang et al. [21] showed that IAUC can successfully differentiate recurrent glioblastoma from treatment-related change. Chung et al. [4] suggested that the IAUC-related parameter can be used to differentiate tumor recurrence from treatment-related change in post-treatment glioblastoma. Kim et al. [15] found the highest diagnostic performance and reproducibility when DCE perfusion MR imaging was combined with contras-enhanced T1-weighted imaging and DWI for differentiating recurrent glioblastoma from treatment-related change. In this study, we found that the model-free DCE parameters could provide an added value to uninterpretable DSC image for differentiating tumor recurrence from post-treatment effect. On the other hand, DWI fails to provide the same level of diagnostic performance as that of DCE perfusion MR imaging in selected patients with uninterpretable DSC perfusion MR images. This can be partly explained by the fact that echo-planar sequence-based DWI is more sensitive to diamagnetic susceptibility artifacts than the gradient-recalled, echo sequence near the skull base, as shown in the previous study [13].

Our study has several limitations. First, current study used the same imaging paramters, MR machine and contrast agents with our previous study [4,15], however the mean IAUC90 values of the two groups and the optimum ROC cutoffs of the IAUC90 for differentiating the two groups in this study were lower than those in our previous studies [4,15]. This discrepancy of the results could have problem in generalization of cutoff value in clinical practice. The different methods for DCE image processing between the previous studies and this study could have partially caused the different results. However, despite considerable variation in both the study patients’ characteristics and the methods of image processing, a similar trend of the positive results could be obtained from the DCE MR studies in patients with post-treatment glioblastoma. Second, our pathologic estimations of recurrent glioblastoma versus treatment-related change were not done by direct lesion-by-lesion analysis. As most of the tumors were intermixed with recurrent tumor and treatment-related change, accurate dichotomization of the diagnosis was inappropriate. Third, per our protocol, dual injections of contrast agent were performed during the combined DCE and DSC MR studies. As in this study, DCE MR perfusion imaging could provide an added value to uninterpretable DSC MR studies, therefore, the simultaneous measurement of DSC and DCE MR imaging parameters might be an efficient means for differentiating tumor recurrence form post-treatment effect in cases with enhancing lesion containg hemorrhagic foci or proximity to the skull base. Lastly, using a model-free DCE parameter, we could not explain other multiple effects, such as edema, which can cause tumor vessel permeability. Although these factors were beyond the scope of this study, the assessment of the other factors will be needed in order to validate our study

## Conclusions

In conclusion, DCE MR imaging could be a superior and more reproducible imaging biomarker than DWI for differentiating tumor recurrence from post-treatment effect in patients with post-treatment glioblastoma when DSC MR images are not interpretable.

## Supporting Information

S1 Fig Comparison of leave-one-out cross-validation. The combination of DCE MR imaging with DWI shows a highest diagnostic accuracy for differentiating tumor recurrence from treatment-related change for both readers.
(TIF)
