# Supplementary figures and images for: Uninterpretable Dynamic Susceptibility Contrast-Enhanced Perfusion MR Images in Patients with Post-Treatment Glioblastomas: Cross-Validation of Alternative Imaging Options
Source: PLoS One. 2015 Aug 21;10(8):e0136380. doi: 10.1371/journal.pone.0136380 (PMC4546423; doi:10.1371/journal.pone.0136380)

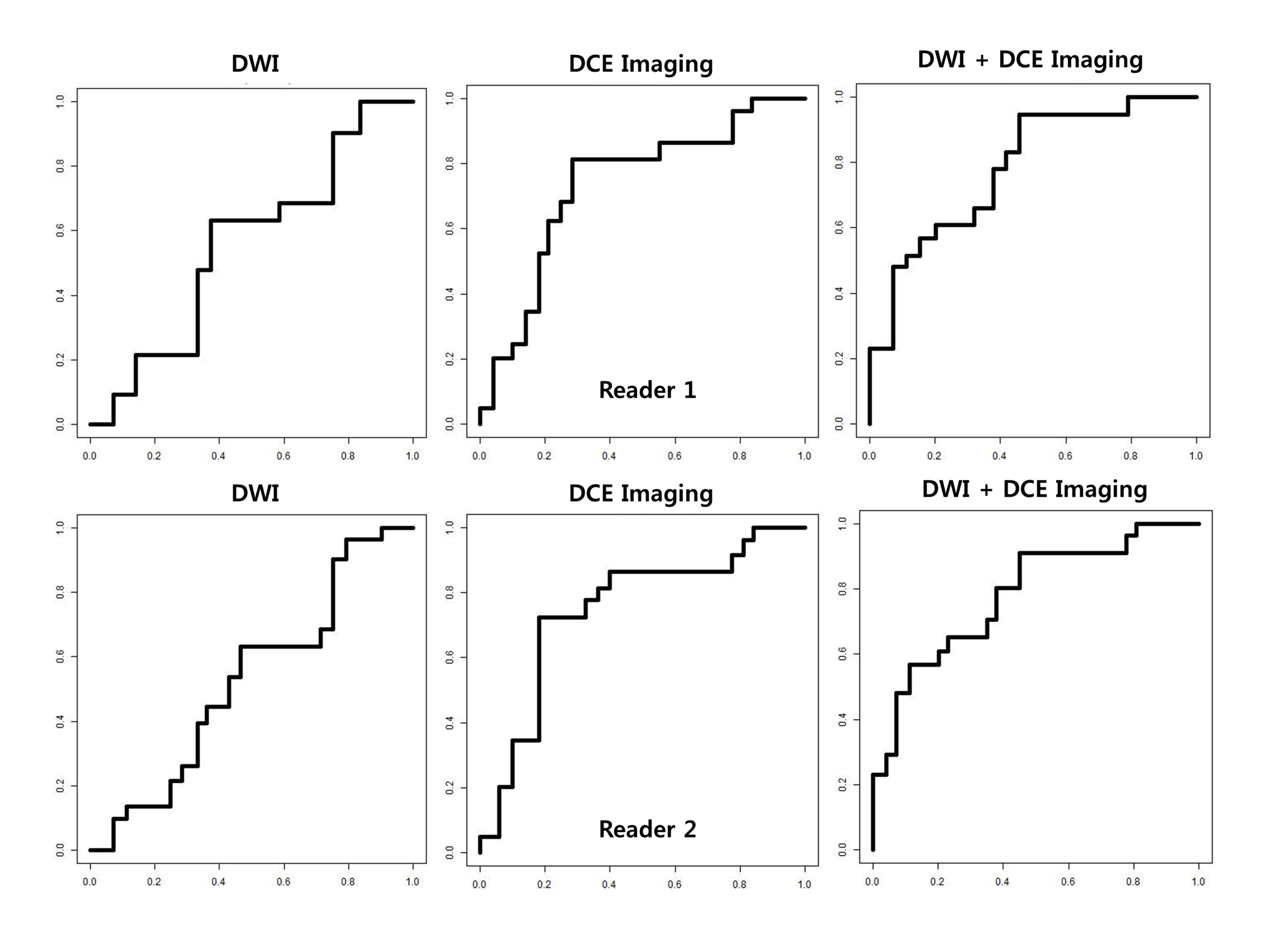

Supplement: S1 Fig — The combination of DCE MR imaging with DWI shows a highest diagnostic accuracy for differentiating tumor recurrence from treatment-related change for both readers. (TIF) [file pone.0136380.s001.tif]
